# Continuous glucose monitoring for automatic real-time assessment of eating events and nutrition: a scoping review

**DOI:** 10.3389/fnut.2023.1308348

**Published:** 2024-01-08

**Authors:** Julian Brummer, Christina Glasbrenner, Sieglinde Hechenbichler Figueroa, Karsten Koehler, Christoph Höchsmann

**Affiliations:** Department of Health and Sport Sciences, TUM School of Medicine and Health, Technical University of Munich, Munich, Germany

**Keywords:** meal detection, continuous glucose monitoring, dietary assessment, healthcare technology, closed loop, sensors, meal timing

## Abstract

**Background:**

Accurate dietary assessment remains a challenge, particularly in free-living settings. Continuous glucose monitoring (CGM) shows promise in optimizing the assessment and monitoring of ingestive activity (IA, i.e., consumption of calorie-containing foods/beverages), and it might enable administering dietary Just-In-Time Adaptive Interventions (JITAIs).

**Objective:**

In a scoping review, we aimed to answer the following questions: (1) Which CGM approaches to automatically detect IA in (near-)real-time have been investigated? (2) How accurate are these approaches? (3) Can they be used in the context of JITAIs?

**Methods:**

We systematically searched four databases until October 2023 and included publications in English or German that used CGM-based approaches for human (all ages) IA detection. Eligible publications included a ground-truth method as a comparator. We synthesized the evidence qualitatively and critically appraised publication quality.

**Results:**

Of 1,561 potentially relevant publications identified, 19 publications (17 studies, total *N* = 311; for 2 studies, 2 publications each were relevant) were included. Most publications included individuals with diabetes, often using meal announcements and/or insulin boluses accompanying meals. Inpatient and free-living settings were used. CGM-only approaches and CGM combined with additional inputs were deployed. A broad range of algorithms was tested. Performance varied among the reviewed methods, ranging from unsatisfactory to excellent (e.g., 21% vs. 100% sensitivity). Detection times ranged from 9.0 to 45.0 min.

**Conclusion:**

Several CGM-based approaches are promising for automatically detecting IA. However, response times need to be faster to enable JITAIs aimed at impacting acute IA. Methodological issues and overall heterogeneity among articles prevent recommending one single approach; specific cases will dictate the most suitable approach.

## 1 Introduction

Nutrition has a major impact on people’s health and well-being (1–8). However, accurately assessing nutrition and dietary intake remains challenging, with the most precise tools often involving high costs, participant and staff burden, or privacy issues (9–14). Yet, valid and reliable measurement of dietary behavior is essential to accurately detect changes in research settings and guide patient counseling in clinical practice (e.g., weight loss programs). Technological advances in recent years have led to new approaches for accurately assessing dietary intake that try to overcome some of the shortcomings of traditional dietary assessment methods (9, 15–18).

An attractive technology-based option for assessing the consumption of calorie-containing foods and beverages (ingestive activity, IA) is continuous glucose monitoring (CGM). CGM involves using a sensor that measures glucose concentrations in the interstitial fluid (19, 20) as a proxy for blood glucose levels (20, 21). CGM has become an important tool in diabetes care (19, 22–25). For instance, it is an integral component of artificial pancreas (AP) systems designed to automate and improve blood glucose regulation in individuals with type 1 diabetes mellitus (T1DM) via the utilization of CGM, an insulin infusion pump, and a control algorithm (26). Beyond diabetes management, CGM is gaining popularity for use in healthy individuals and athletes (20, 27). Several CGM devices show satisfactory accuracy data (20, 28, 29).

The automatic and (near-)real-time detection of IA via CGM could offer benefits in (clinical) practice, including a reduced participant and staff burden. In addition, interventionists could monitor meal plan adherence more closely and detect deviations from intervention goals as they occur. Consequently, targeted and personalized countermeasures could be deployed proactively. One particularly useful approach would be CGM-based detection of IA in the context of Just-In-Time Adaptive Interventions (JITAIs). JITAIs aim to exploit the full potential of remote monitoring combined with delivering intervention content in the moment/context when it is most needed and the patient is likely to be (most) receptive (30). Preliminary results show promising effects of JITAIs on predicting and preventing dietary lapses (31). If detection times of the CGM-based approaches in question were extremely short (e.g., less than a few minutes), JITAIs could aim at acutely impacting IA (e.g., sending a prompt asking a person to terminate a meal). If detection times were relatively short (e.g., less than an hour), JITAIs could aim at altering subsequent IA (e.g., a dinner meal). In both cases, information on IA would be much more readily available than with traditional dietary assessment methods (e.g., 24-h recalls).

However, there may also be challenges associated with the use of CGM for the automatic monitoring of IA. On the one hand, there are system-inherent challenges. For example, postprandial rises in blood glucose vary in timing and extent depending on meal composition, meal quantity, inter-individual variability, and many other factors (32–40). Further, there is a delay between interstitial fluid and blood glucose concentrations (20, 41, 42). On the other hand, blood glucose levels are not only influenced by IA but also by other factors such as physical activity, stress, and diurnal fluctuations (20, 41, 43–55). Thus, false positive detections (e.g., erroneously flagging a meal due to glucose increases caused by stress) and false negative detections (e.g., erroneously *not* flagging a meal because other factors render the glucose response too flat) might occur.

In the past years, research examining the use of CGM for the automatic detection of IA has accumulated. Recent publications reviewed options for the automatic detection of IA using wearable−/sensor-based methods (15–18), but they did not specifically address CGM. The present article aims to close this research gap and answer the following guiding questions:

1. Which approaches using CGM for the automatic detection of IA in (near-)real-time have been investigated, and have these approaches relied solely on CGM or also used other data (e.g., sensors/wearables)?
2. How accurate are these approaches in detecting IA?
3. Can these approaches be used in the context of JITAIs?

## 2 Methods

The reporting of this review is based on the updated Preferred Reporting Items for Systematic Reviews and Meta-Analyses Extension for Scoping Reviews (PRISMA-ScR) guideline (56).

### 2.1 Search strategy

The primary systematic literature search was conducted on 09 September 2022, using the IEEE Xplore, PubMed, Scopus, and Web of Science databases. An identical supplementary search was conducted on 02 October 2023. The search term was developed and refined by two authors (JB, CH) to capture all relevant publications, and the search term contained: (intake OR uptake OR eating OR ingest* OR meal OR drink* OR beverage OR consum* OR oral) AND (monitor* OR assess* OR detect* OR estimat* OR measur* OR sens*) AND (“continuous glucose monitoring” OR “real time continuous glucose monitoring” OR “real-time continuous glucose monitoring” OR “flash glucose monitoring” OR “intermittently scanned continuous glucose monitoring” OR CGM OR rtCGM OR isCGM OR “artificial pancreas” OR “artificial beta cell*” OR “artificial beta-cell*” OR “artificial β-cell*” OR “artificial β cell*”) AND (algorithm OR “deep learning” OR “machine learning” OR “neural network*” OR AI OR “artificial intelligence“). Because a fully-closed-loop AP system must first detect meals to adequately manage the following increases in glucose by delivering insulin to the patient (57), the search also included AP systems.

JB conducted the database searches and removed duplicates for the primary and supplementary searches. Two authors (JB, CH) screened the titles and abstracts against the predefined eligibility. In discrepancies, a consensus was reached via discussions and ineligible publications were discarded. For the primary search, JB screened the full texts of the remaining publications for eligibility and consulted with CH, who then independently screened these full texts in cases of uncertainty. In addition, one other author (SHF) conducted independent cross-checks for a randomly selected 20% of the full texts. For the supplementary search, two authors (JB, CH) independently screened the full texts of the remaining publications. Again, in case of discrepancies, a consensus was reached via discussions, and subsequently, ineligible publications were discarded. Another author (CG) was consulted for her technical/mathematical expertise during the screening process. JB hand-searched the reference lists of eligible publications for any additional relevant literature. In several cases, the corresponding authors of articles were contacted, e.g., to receive full texts or raw data or to clarify results.

### 2.2 Eligibility criteria

We included publications if the following inclusion criteria were met: (1) publications were written in English or German and published until 2 October 2023; (2) publications are original articles published in peer-reviewed scientific journals or conference papers; (3) at least one performance measure of the automatic detection of IA is reported explicitly. For example, the accuracy was calculated by comparing the CGM-*based* (did not have to exclusively rely on CGM as input) approach against a ground-truth method (e.g., self-reported or observed IA); (4) a CGM-based approach was used to detect IA *in vivo* in free-living, semi free-living, or laboratory settings. This also included trials of AP systems if criterion 3 was met; (5) only the most recent publication on a specific approach by a particular research group was included if it supersedes preceding publications.

We excluded publications for the following exclusion criteria: (1) the approach was not tested in human participants (e.g., *in silico* studies); (2) no outcome results were reported (e.g., study protocol publications); (3) outcomes did not include an explicit performance measure describing the results of the automatic detection of IA (e.g., only figures showcasing the CGM trends over time); (4) the methodology was described without sufficient detail. We did not apply restrictions regarding publication date or participant age.

### 2.3 Data extraction

The following information was extracted: (1) first author and publication year; (2) a summary of the study; (3) sample size and, if available, sex and age of participants; (4) participants’ diabetes status [no diabetes, prediabetes, T1DM or type 2 diabetes mellitus (T2DM)]; (5) scope of the study (duration/number of IA events) and, if available, information on the IA events (e.g., meal composition); (6) ground-truth/criterion method(s); (7) performance measure(s); (8) details on the CGM device and if applicable other relevant devices used in the study.

JB extracted relevant information from the original publications, and in cases of uncertainty, the respective publications were double-checked by CH. Two other authors (CG, SHF) also double-checked the extracted information. One other author (CG) further extracted technical details of the tested approaches.

### 2.4 Data synthesis

We synthesized the evidence qualitatively, focusing on answering the three research questions outlined above. Although using an explicit cutoff (e.g., ≥80% F1-score or accuracy) is desirable for performance evaluation and has been used in a related review (16), this approach was not feasible, as only a few publications reported accuracy and/or F1-score values.

Furthermore, we appraised the included publications critically. We considered the following aspects of being of concern: (1) error-prone methods for identifying the ground truth of IA [e.g., self-reported IA (58) or retrospective identification from CGM data, whereas inpatient settings with observed IA were generally assumed to be less error-prone]; (2) a sample consisting exclusively of individuals with diabetes as this might limit generalizability to non-diabetic populations; (3) meal announcement/meal-accompanying insulin boluses, as there might be an interference with the (early) postprandial blood glucose levels that are relevant for the automatic detection of IA; (4) algorithm inputs other than CGM since ultimately a CGM-only approach would be desirable to minimize costs and effort.

## 3 Results

**Figure 1:**
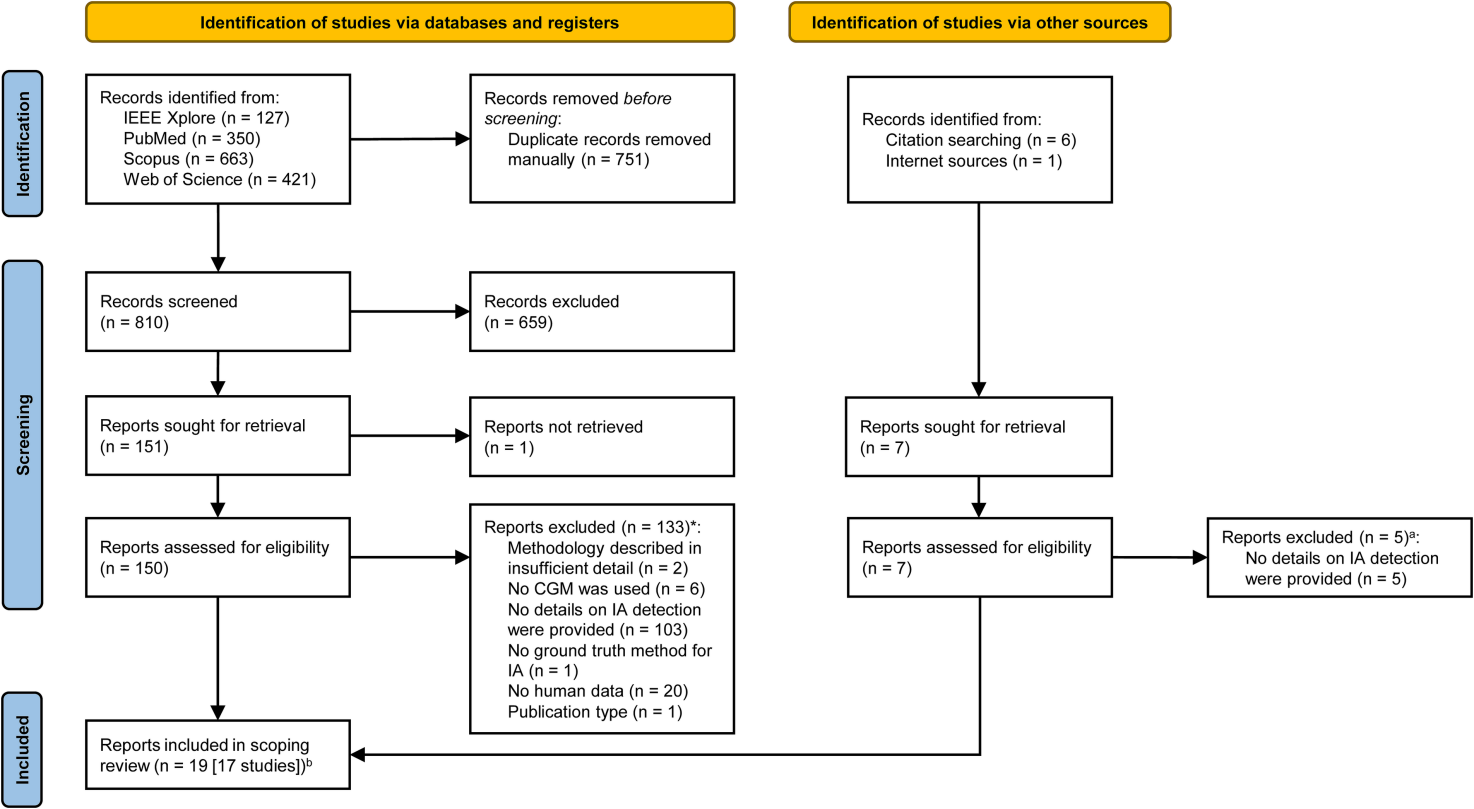
PRISMA Flow diagram (88). ^a^In some cases, more than one reason led to the exclusion of a publication; here, only the primary reason is listed for each publication. ^b^In two cases, two publications of the same study were included. CGM, continuous glucose monitoring; IA, ingestive activity.

The literature search identified a total of 1,561 potentially relevant publications. Nineteen publications reporting data from 17 studies (for 2 studies, 2 publications each were relevant, see Table 1), including 311 participants, met the inclusion criteria (59–66, 68, 70, 71, 73–75, 82–85, 87). Figure 1 shows the process of the literature search, screening, and selection in a PRISMA-style flow diagram (88).

**Table 1:** Characteristics of included publications.

Author, year	Study summary	*N* (male/female), age (if available)	Diabetes status	Scope of the study/information on the assessed eating events	Ground-truth method(s)	Performance measure(s)^a^	Details on the CGM device and other input devices
Atlas et al. (59)	Pilot feasibility clinical trial investigating the performance of the MD-Logic Artificial Pancreas (MDLAP) System, a fully CL system utilizing a patient’s diabetes treatment management in conjunction with a fuzzy logic-based control-to-range module and a control-to-target module to control blood glucose levels.	7 (2/5); 23.9 ± 3.4 years, range 19–30 years	T1DM	8 h CL sessions in a resting state, with 9 fasting sessions (*n* = 6) and 3 meal challenge sessions (*n* = 2; mixed meal with 40–60 g of CHO after 8 h fast)	Inpatient study	Overall mean detection time: 23 min after consumption	CGM: Freestyle Navigator (Abbott Diabetes Care, Alameda, CA) or STS-Seven System (DexCom, San Diego, CA); sampling rate: 5 min
				Two participants completed 1 additional 24 h CL session each, in which mixed meals (CHO content for each meal was 17.5–70 g) were consumed at 1,930 h, 0800 h, and 1,300 h, with participants entering the sessions after ≥3 h of fasting.			
Bertrand et al. (60)^b^	Comparison of eating activity detection systems using (1) wearable wristbands vs. (2) wearable wristbands + CGM. Three machine learning algorithms were applied for the classification of eating and non-eating events: support vector machine (SVM), random forest (RF), and extreme gradient boosting tree (XGB). For each algorithm, one model based on the CGM data was compared to a model without CGM data.	10 (5/5); range 19–51 years	Healthy, non-diabetic	Up to 2 weeks	An app (“aTimeLogger”) was used to log the ground truth	Mean (standard deviation)^c^:	CGM: FreeStyle Libre 2
						MCC_XGB_: 0.35 (0.10) − MCC_SVM_: 0.37 (0.06)	
						F1-score_XGB_: 0.49 (0.10) − F1-score_SVM_: 0.50 (0.08)	
						Sensitivity_XGB_: 0.67 (0.19) − Sensitivity_SVM_: 0.63 (0.20)	Wearable wristbands measuring steps and heart rate: Fitbit Charge 3 (dominant hand) and Mi Band 4 (non-dominant hand)
						Specificity_XGB_: 0.74 (0.07) − Specificity_SVM_: 0.77 (0.14)	
						Precision_XGB_: 0.41 (0.10) − Precision_SVM_: 0.46 (0.11)	
Bertrand et al. (61)^b^	This publication used the same dataset as Bertrand et al. (60). Two tree-based ensemble learning algorithms were used: random forest (RF) and extreme gradient boosting tree (XGB). Compared to Bertrand et al. (60) different resampling techniques were investigated for their performance in detecting eating activities vs. non-eating activities: no resampling (-N), random up-sampling (-U), random down-sampling (-D), and SMOTE resampling (-S).The combination of the two machine learning algorithms and the different resampling techniques resulted in eight classification models that were compared.	10 (5/5), average age: 32 years	Healthy, non-diabetic	Up to 14 days; in total, 1,361 activity events were collected	Free-living environment ➔ participants used an app (“aTimeLogger”) to log the ground truth of their activities	Mean (standard deviation)^d^:	CGM: a FreeStyle Libre 2
						MCC_XGB-N_: 0.34 (0.13) − MCC_XGB-U_: 0.38 (0.12);	
						F1-score_XGB-N_: 0.33 (0.16) − F1-score_RF-S_: 0.51 (0.10);	Wearables: Mi Band 4 (non-dominant wrist), FitbitCharge 3 (dominant wrist)
						Sensitivity_XGB-N_: 0.23 (0.14) − Sensitivity_XGB-D_: 0.67 (0.19);	
						Specificity_XGB-D_: 0.74 (0.07) − Specificity_XGB-N_: 0.98 (0.03);	
						Precision_XGB-D_: 0.41 (0.10) − Precision_XGB-N_: 0.80 (0.21);	
						Accuracy_XGB-D_: 0.73 (0.03) − Accuracy_XGB-N_: 0.82 (0.05).	
						RF-S was deemed to have the best overall performance of the eight models.	
Dassau et al. (62)	Using data from a pilot study, four different detection methods were compared: backward difference (BD), Kalman filter estimation (Kalman), combination of BD and Kalman (BD + Kalman), and second derivative of glucose (G“). Central aim was to reduce FP detections to reduce the risk for erroneous insulin injections in the context of an AP. To do so, a voting algorithm was implemented, using either a two-out-of-three (BD, BD + Kalman, and G”) or three-out-of-four (BD, Kalman, B + Kalman, and G”) scheme to check for concordance in meal detections by the above-mentioned methods. Importantly, insulin meal boluses were purposefully delayed by 1 h and thus did not confound the postprandial BG changes.	17 (40% girls); 11 ± 4 years, range 4–17 years	T1DM	17 breakfast meals (one per participant) with an average of 56 g of CHO (range: 22–105 g of CHO), the content of which was decided upon by the participants	Inpatient study	ΔT (min) = Detection time from the onset of the meal; ΔG (mg/dL) = Difference in the glucose level when detection took place minus the preprandial value.	CGM: FreeStyle Navigator (Abbott Diabetes Care, Alameda, CA) with a sampling rate of 1 min
						Average values for the four different detection methods: ΔT_BD_ = 29 min, ΔG_BD_ = 13 mg/dL; ΔT_Kalman_ = 35 min, ΔG_Kalman_ = 30 mg/dL; ΔT_BD + Kalman_ = 31 min, ΔG_BD + Kalman_ = 18 mg/dL; ΔT_G”_ = 30 min, ΔG_G”_ = 16 mg/dL.	
						ΔG using the Kalman algorithm was statistically significantly higher compared with ΔG using the other methods (*p* < 0.001).	
						Average values for the different voting schemes: ΔT_two-out-of-three_ = 30 min, ΔG_two-out-of-three_ = 15 ± 10 mg/dL; ΔT_three-out-of-four_ = 32 min, ΔG _three-out-of-four_ = 21 ± 9 mg/dL.	
Dovc et al. (63)	Double-blind, randomized, two-period crossover study on the safety and efficacy of fully CL insulin therapy/glucose control using two different insulin solutions (faster vs. standard insulin aspart). Atlas et al.’s (59) fuzzy logic-based control algorithm was used (see above).	20 (9/11); 21.3 ± 2.3 years	T1DM	Two 27-h (1,500 h on the first day to 1,800 h the next day) CL inpatient stays with meals that were unannounced to and thus uncovered by the fully CL device. Standardized and identical meals were given on both study visits. Main meals contained ~1 g of CHO/kg of body mass and snacks about half of this amount. Macronutrient distribution was about 50% CHO, 20% proteins, and 30% fats (<10% saturated fats). Meals were scheduled at: ~1,500 h (snack); 1 h after the end of an exercise protocol between 1,900 and 2,000 h (dinner); 0800 h (breakfast); 1,200 h on the next day (lunch).	Inpatient study	Median time of delivered prandial bolus was 38.4 min (32.7, 55.8) for meals in the faster insulin aspart arm and 30.1 min (26.9, 54.6) in the standard insulin aspart arm (*p* = 0.388).	CGM: Enlite II sensor (Medtronic Diabetes); CL algorithm: DreaMed GlucoSitter (DreaMed Diabetes, Petah Tikva, Israel)
El Fathi et al. (64)^e^	Preliminary results from a randomized three-way experiment on the safety and efficacy of CL insulin delivery with or without a meal detection module (an adaptive model-based meal detection algorithm) versus conventional pump therapy after a missed insulin bolus. The data stem from the same study as Palisaitis et al. (65).	4 adolescents	T1DM	Per participant, three 9-h inpatient visits with one uncovered lunch meal with 60 g of CHO per visit were conducted ➔ 4 participants x 3 visits x 9 h = 108 h of data	Inpatient study	Comparison of the incremental AUCs after the missed insulin bolus across the three conditions: conventional pump therapy = reference standard (29.6 ± 6.5 h mmol/l), CL without meal detection: −16% incremental AUC (24.8 ± 11.5 h mmol/l), CL with meal detection: −39% incremental AUC (18.0 ± 2.7 h mmol/l); The collected data were also used to run the meal detection algorithm offline over the 108 h (4 patients × 3 visits × 9 h) of clinical data: 12/12 unannounced meals detected successfully; no FPs; time until meal detection = 35 [30–40] min; glucose increase at meal detection time = 2.89 ± 1.72 mmoL/L	NIA
Faccioli et al. (66) [supplemented with information from Anderson et al. (67)]	Data from a multicenter clinical trial on the feasibility of a long-term automated insulin delivery system were used for a retrospective evaluation of a super-twisting-based meal detector. 14 days of SAP therapy under free-living conditions preceded the main phase of the study; these data were used for the evaluation of the meal detector. Due to the use of SAP therapy, manual meal insulin boluses were given, and the results need to be interpreted taking this into consideration.	30 (17/13); median age 44 years, range 18–66 years	T1DM	14 days	Patient-reported meal times. Of note, 11/30 participants had <20 registered meals for 14 days. Since some missed meal announcements occurred, the authors only selected portions of data with certain meal information.	All values refer to median (interquartile range): TP = 16 (10); FN = 6 (4); FP = 7 (3); Recall = 70% (13%); Precision = 73% (26%); F1-score = 68% (16%); FP per day = 1.4 (1.4); CHO content related to FNs = 32 g (32 g); detection time = 45 min (45 min)	CGM: DexCom G4 (DexCom, Inc., San Diego, CA, United States); sampling time of 5 min
Fushimi et al. (68) [supplemented with information from Sánchez-Peña et al. (69)]	CGM data obtained during a clinical trial were used to evaluate the Automatic Regulation of Glucose (ARG) algorithm with an additional automatic switching signal generator (SSG), i.e., a meal detection module. Importantly, in the clinical trial meal announcement was used, so the results need to be interpreted in light of this potential bias.	5 (2/3), 43 ± 6 years, range 32–48 years	T1DM	Five standardized meals per participant: one breakfast, one lunch, one afternoon snack, two dinners. Breakfast and afternoon snack: a cup of tea or coffee, two slices of whole-meal bread or five crackers, diet jam, spreadable cheese (≈28 g of CHO). Dinners: whole pasta, lean meat, fresh fruit (≈55 g of CHO). Lunch: same as dinners, but mashed potatoes instead of whole pasta (≈55 g CHO). One meal was excluded due to pump occlusion ➔ 24 eligible meals	Inpatient study	2 FPs (8.3%); 2 FNs (8.3%); efficiency = 83.3%	CGM: Dexcom G4 sensor (Dexcom, San Diego, CA), sampling rate: 5 min
Godoy et al. (70)	A feedback scheme-based meal detection and CHO estimation algorithm was developed and evaluated retrospectively on a clinical dataset.	11 adults	T1DM	5 days, whereby the first 3 days were used for identification/calibration and the following 2 days were used for the validation of the proposed model	Free-living data with CGM measurements, insulin pump recordings, participant-recorded CHO estimates, etc.	184 TPs; 263 TNs; 9 FPs; 2 FNs; 98.92% sensitivity; 96.69% specificity; 97.60% accuracy^f^; Mean time gap (estimated meal onset time – actual meal onset time) = 9.0 min and 25 min delay time	Insulin pump MiniMed 640G; CGM sampling time = 5 min, but up-sampled to 1 min to increase the detection sensitivity
	Of note, insulin boluses were used as another algorithm input. The results need to be interpreted considering this.						
Hoyos et al. (71) [supplemented with information from Aleppo et al. (72)]	Data from a study assessing the reliability of CGM measurements were used to compare two scenarios: one scenario with the original meal events announced by the participants and one with the meal events generated automatically by the super-twisting-based meal detector introduced in Faccioli et al. (66). “An unsupervised clustering algorithm based on Fuzzy C-Means was applied to classify event-to-event segments of CGM data. Events defining data partitioning were automatically generated based on: (1) an automatic meal detection algorithm (for day periods) and (2) time of day (for night periods).” (p. 576)	44 adults	T1DM	26-week study; only participants with an average of 3 to 5 reported meals per day were considered	Free-living data with CGM measurements, insulin pump recordings, etc.	Results (M ± SD) for automatically detected meals: Number of clusters (c*) = 8.09 ± 1.67; Fukuyama-Sugeno index (V_FS_) = −16,893 ± 5,838; Compactness (V_com_) = 0.236 ± 0.063; Variance (V_var_) = 966 ± 653.4; Time in range = 45.2 ± 15%	CGM: Dexcom G4 Platinum; Sampling time = 5 min
						There was lower variance in the clusters of the automatically detected meals as compared to the announced meals, thereby underscoring the algorithm’s value.	
Kölle et al. (73)	Four different detection methods were retrospectively compared using a clinical dataset: two methods based on the classification of horizons (classification of estimated R_α_ horizons [LDA R_α_] and classification of CGM horizons [LDA CGM], respectively) and two methods based on threshold violations (threshold on current R_α_ estimate [Threshold] and the previously published Glucose Rate Increase Detector algorithm (79), respectively). Note that often meals were accompanied by insulin boluses, so, again, results need to be interpreted in light of this.	12 (8/4); 7.3 ± 4.7 years	T1DM	492 of 849 identified meals were included, whereby the authors focused on meals that would necessitate automatic meal detection (e.g., larger meals); meals were divided into categories of pre-meal, at-meal, post-meal and no insulin bolus	Two experienced diabetologists independently retrospectively identified meals from free-living CGM data and logged information from insulin infusion pumps.	Averages across 10 cross-validated Monte Carlo runs:	CGM: Medtronic Enlite 2
						LDA R_α_: sensitivity = 0.92; 1.50 FPs/day; time of detection after meal start = 18.59 min	
						LDA CGM: sensitivity = 0.90; 1.37 FPs/day; time of detection after meal start = 11.78 min	
						Threshold: sensitivity = 0.64; 1.28 FPs/day; time of detection after meal start = 32.67 min	
						GRID: sensitivity = 0.21; 2.81 FPs/day; time of detection after meal start = 42.53 min	
Mosquera-Lopez et al. (74)	A single-center, randomized crossover trial was conducted to compare postprandial (4 h) glucose control following unannounced meals using a hybrid model predictive control algorithm and a newly developed robust artificial pancreas (RAP) system (i.e., two intervention visits per participant). The RAP system used machine learning for automated meal detection and meal size estimation. CGM and insulin data were used.	15 (6/9) participants enrolled (age: 37.6 ± 10.4 years), 2 participants withdrew from the study ➔ 13 included in analysis	T1DM	Only the intervention visit involving the RAP system was used to determine meal detection performance; this visit involved a total of 24 participant-chosen study meals with a CHO content of 45-66 g.	Participant confirmation of alerts sent out by the meal detection system; data on mealtime records entered into a cloud-based database by a study investigator.	Sensitivity = 83.3% (95% CI 62.6–95.2%); false discovery rate = 16.6% (95% CI 4.7–37.4%); detection time (M ± SD) = 25.9 ± 0.9 min	CGM: Dexcom G6 (DexCom, Inc., San Diego, CA, United States); Insulin pump: Omnipod (Omnipod Insulet Corporation, Acton, MA, United States)
Ornetzeder et al., 2019 (75) [supplemented with information from Zschornack et al. (76)]	Three previously published meal detection algorithms (79–81) were compared by using data from two clinical trials, one with participants with T1DM and one with insulin-treated participants with T2DM. Importantly, in both datasets insulin meal boluses were given, so the results need to be interpreted in light of this. Furthermore, small meals (<20 g of CHO) were treated differently from larger meals in that they did not contribute to FN and FP counts.	10 ➔ 5 (3/2) participants with T1DM (mean age: 48 years) and 5 (4/1) participants with T2DM (mean age: 65 years); both samples were random subsamples of the respective study samples	T1DM, T2DM	T1DM participants: datasets with 7 days per participant but the first 48 h after the insertion of the CGM sensor were not considered for the performance assessment; T2DM participants: datasets with 2–3 days per participant	T1DM: no further information	T1DM (fixed parameters; all means): ΔT_Harvey_ = 19.1 min; ΔT_Samadi_ = 12.7 min; ΔT_Ramkissoon_ = 19.6 min; FP/day_Harvey_ = 0.6; FP/day_Samadi_ = 0.9; FP/day_Ramkissoon_ = 0.4; Sensitivity_Harvey_ = 77.0%; Sensitivity_Samadi_ = 73.7%; Sensitivity_Ramkissoon_ = 75.0%	T1DM: prototype CGM system using a sensor in an early development phase (Roche Diagnostics GmbH, Mannheim, Germany)
					T2DM: outpatient study	T2DM (fixed parameters; all means): ΔT_Harvey_ = 27.8 min; ΔT_Samadi_ = 24.6 min; ΔT_Ramkissoon_ = 26.9 min; FP/day_Harvey_ = 1.3; FP/day_Samadi_ = 1.3; FP/day_Ramkissoon_ = 0.9; Sensitivity_Harvey_ = 70.5%; Sensitivity_Samadi_ = 67.9%; Sensitivity_Ramkissoon_ = 70.7%	
						T2DM (patient-specific parameters; all means): ΔT_Harvey_ = 30.7 min; ΔT_Samadi_ = 30.5 min; ΔT_Ramkissoon_ = 26.1 min; FP/day_Harvey_ = 1.0; FP/day_Samadi_ = 1.1; FP/day_Ramkissoon_ = 0.4; Sensitivity_Harvey_ = 79.9%; Sensitivity_Samadi_ = 80.2%; Sensitivity_Ramkissoon_ = 67.3%	T2DM: CGM sampling rates: 1 min and 5 min for the T1DM and T2DM datasets, respectively
					For both trials CGM data and information on the ingested amount of CHO, meal timing, and insulin were recorded	ΔT was defined as the time between meal ingestion and the detection event	
Palacios et al. (82)	Data was collected in a cross-over study on post-resistance exercise nutrient timing comparing immediate vs. 3-h delayed post-exercise nutrition. Tree-based machine learning models (random forest model and gradient boosting machines) using a cold-start and a non-cold-start approach were applied, respectively. Six primary variables were used for the machine learning model: glucose, heart rate, physical activity, core temperature, skin temperature, and respiration rate. As physical activity was found to not aid in prediction it was removed and new lag variables were included.	9 (9/0); 24.3 ± 4.6 years	Healthy, non-diabetic	48 h; Total daily energy intake had an approximate macronutrient distribution of 52% CHO, 32% fat, 16% protein; meals were consumed at approximately 08:00, 11:20, 16:00, and 18:00 on both days.	48-h inpatient study in a whole-room calorimeter ➔ exact mealtimes were recorded	(1) area under the receiver operating characteristic curve (AUC-ROC); (2) area under the precision-recall curve (AUC-PR) ➔ cold-start: *k* = 110 min: AUC-ROC = 0.891; AUC-PR = 0.803; non-cold-start: *k* = 20 min, AUC-ROC = 0.996; AUC-PR = 0.964	CGM: iPRO Professional CGM System (Medtronic MiniMed, Inc., Northridge, CA) placed on the abdomen; Equivital Sensor Electronics Module (Equivital I: Hidalgo Ltd., Cambridge, United Kingdom) combined with a heat-sensitive transmitter (pill) to assess heart rate, heat flux, core body temperature; To match the calorimeter data, the Equivital and CGM data were resampled using cubic spline interpolation
						*k* = minimal window size	
Palisaitis et al. (65)^e^	Randomized, three-way, crossover trial comparing an AP system equipped with a meal detection algorithm (AP + MDA) with the AP alone and conventional pump therapy (CSII) in controlling blood glucose levels after a meal without accompanying insulin bolus. The data is from the same study as El Fathi et al. (64).	11; 14.9 ± 1.3 years	T1DM	One self-selected lunch meal which was standardized between interventions for each participant ➔ Mixed meals with 55-65 g of CHO served 4 h after the start of the intervention	Inpatient setting or at home with a member of the research staff with three 9-h interventions from 0800 to 1,700 or 0900 to 1,800	Median meal detection time in the AP + MDA condition: 40.0 min (interquartile range 40.0–57.5 min) after consumption of the meal; Incremental glucose from the start of the meal until time of meal detection: 2.6 mmol/L [2.4–4.8], and a rate of change of 10.1 [7.3–12.5] mmol/L/h	CGM: Dexcom G5
Popp et al. (83)	Self-reported (SR; using a smartphone app) timing of eating occasions (consumption of foods and beverages >0 kcal) was compared to objective assessment methods, i.e., a wrist-motion-based (WM) classifier using an ActiGraph worn on participants’ dominant wrists and a simulation-based explanation system using CGM. The data come from an ancillary study of a weight loss intervention study.	31 completers; 62% females; age: 59 ± 11 years	Prediabetes/moderately controlled T2DM	10 days	Free-living; Date- and time-stamped eating occasions were entered into a smartphone app; herein, we assume that these serve as the ground-truth method.	CGM method found the longest eating window and the largest number of eating occasions per day.	ActiGraph: ActiGraph GT9X-BT (Pensacola, FL, United States).
						Pearson’s correlations: first eating occasion identified by SR and CGM: r = 0.534, *p* = 0.01; first eating occasion identified by CGM and WM: r = 0.325, *p* = 0.004; eating midpoint identified by CGM and WM: r = 0.253, *p* = 0.03.	
						Overlap between methods: Tolerance windows of ±0, 5, and 10 min: <40% of eating occasions identified by both WM and CGM; tolerance windows of ±30, 60, 120 min: overlap between SR and CGM: 55 to 80% of eating occasions; overlap between WM and CGM: ~23% regardless of the tolerance window used.	CGM: Abbott Freestyle Libre Pro (Abbott Park, IL, United States) providing 15-min average glucose values
						% of meals identified by all methods: Tolerance windows of ±0, 10 and 15 min: no matching meals identified by all methods; tolerance window of 30 min: 4% of SR meals were also detected by CGM and WM; tolerance window of both 60 and 120 min: 7% overlap; overlap of the three methods over 3 days was found in only one participant	
Samadi et al. (84)	Retrospective evaluation of meal detection and CHO estimation algorithms using clinical data collected in CL experiments using the integrated multivariable adaptive AP system (IMA-AP). Their approach relies on the qualitative analysis of the glucose trajectory and preceding insulin infusion data to detect disturbances and estimate their magnitude by estimating the amount of ingested CHO. Importantly, in these experiments, no meal announcement-based feedforward meal bolusing was used, so the data do not include manual meal-time insulin boluses.	11; 18–35 years	T1DM	117 meals/snacks (7–9 meals and a maximum of 6 snacks per participant) which are distinguished by a CHO threshold of 35 g	NIA	Detection rates (sensitivity): 93.5% (86/92) for meals and 68.0% (17/25) for snacks; FP rate 20.8% (27 FPs and 103 TPs); this equates to 1.05 FPs per day.	CGM: sampling time: 5 min
						Higher probability of detection with higher CHO contents.	
						For detected meals and snacks the increase in glucose from consumption until detection is on average 8.8 ± 21.3 mg/dL (in median ± mean absolute deviation [MAD] as 10.0 ± 14.4 mg/dL) for detected meals and snacks	Real-time data on biometric variables: BodyMedia SenseWear armband and Zephyr chest-band (Bioharness-3; Zephyr Technology, Annapolis, MD)
						Detection time (time from start of the meal to when the algorithm first reports a CHO estimate): 34.8 ± 22.8 min (in median ± MAD as 30.0 ± 16.0 min).	
Turksoy et al. (85) [supplemented with information from Turksoy et al. (86)]	Data obtained during AP trials without meal announcement were used to test a new meal detection approach that requires only CGM data. The meal detection algorithm was meant to be integrated into the integrated multivariable adaptive AP (IMA-AP).	9 (9/0); mean age 18.3 years	T1DM	32-h CL sessions were conducted with each participant including breakfast, lunch, dinner and a snack as well as additional snacks if requested by participants; 63 dietary events (50 main meals and 13 snacks) ➔ Foods were selected based on subjects’ personal requirements and there was no limitation on food intake; M = 44 ± 9.38 g of CHO, whereby main meals were higher in CHO than the snacks.	Inpatient study	61/63 (96.8%) meals/snacks detected successfully; 2 FNs; 1 FP; For the events that were detected successfully the average change in glucose from the start of the meal until the time of the meal detection is 16 ± 9.42 mg/dL.	CGM: Guardian REAL-time CGM (Medtronics, Northridge, CA, United States); sampling time of 5 min, but interpolations used to generate 1-min sampled data
Weimer et al. (87)	Evaluation of a physiological parameter-invariant (PAIN)-based meal detector against three established meal-detection algorithms (62, 79) (89) on a clinical dataset. Importantly, participants used insulin therapy during the monitoring period.	61; 45.7 ± 15.3 years	T1DM	Average duration of monitoring: 17 days	Patient-reported mealtimes (time of inputting meal information into the insulin pump)	Detection rate (i.e., correctly detecting meals within 2 h of the patient-reported mealtime) of the detectors based on operating points that are closest to 2 FPs/day (i.e., relative sensitivity for a standardized specificity): PAIN = 86.9% at 2.01 FPs/day; Dassau et al. = 74.1% at 1.99 FPs/day; Lee & Bequette = 73.4% at 1.99 FPs/day; Harvey et al. = 79.4% at 1.97 FPs/day	CGM: 5-min CGM readings
						With detection rates ≥ 55% and ≤3.7 FPs/day the PAIN-based detector performs more reliably across individuals than the other approaches (based on *n* = 53 since participants with <10 reported meals were excluded from this analysis). The other approaches show lower detection rates (on average and worst-case). The other approaches have lower average FP rates but display greater variance and higher FP rates in the worst-case scenarios.	

Publications included in the review, ordered by the first author’s name. Some publications also include further information (e.g., in silico validation data, CHO content estimation), which are not discussed here. ^a^Only performance measures relevant for our research questions are reported herein. ^b^Bertrand et al. (60) and Bertrand et al. (61) are reports of the same study. ^c^The authors of this paper report many different performance metrics for each algorithm; for space-saving reasons we only report the lowest and highest values for each performance metric here. These values refer to the models that included CGM data. ^d^The authors of this paper report many different performance metrics for each algorithm; for space-saving reasons we only report the lowest and highest means (and the according standard deviations) for each performance metric here. ^e^El Fathi et al. (64) and Palisaitis et al. (65) are reports of the same study. ^f^We calculated sensitivity, specificity, and accuracy from the total number of TPs, TNs, FPs, and FNs, respectively, and thus, the values refer to the total sensitivity, specificity, and accuracy across the sample. AP, artificial pancreas; AUC, area under the curve; BG, blood glucose; CGM, continuous glucose monitoring; CHO, carbohydrates; CI, confidence interval; CL, closed-loop; CSII, continuous subcutaneous insulin infusion; FN(s), false negative(s); FP(s), false positive(s); LDA, linear discriminant analysis; M, mean; MCC, Matthew’s correlation coefficient; NIA, no information were available; SAP, sensor-augmented pump; SD, standard deviation; T1DM, type 1 diabetes mellitus; T2DM, type 2 diabetes mellitus; TN(s), true negative(s); TP(s), true positive(s).

Many of the screened publications were excluded from the present review because they did not include details on the detection of IA but instead focused on measures of glycemic control (e.g., time in specific glucose ranges). Further, some publications were excluded because they included graphic CGM data with IA marked as such but did not provide quantitative data on the detection of IA. Another common reason for exclusion was investigation *in silico*, often using virtual patients with T1DM.

We were unable to retrieve the full text of one publication despite several efforts to contact the authors directly. This publication was excluded; however, it was considered likely ineligible based on its abstract.

### 3.1 Study characteristics

The included publications were published between 2008 and 2023 and reported an average sample size of 18.3 (*SD* = 15.1) participants. Table 1 provides an overview of the included publications and the extracted information. The publications covered a wide age range, including pediatric (62, 73), adolescent (62, 64, 65), and adult (59–61, 63, 66, 68, 70, 71, 74, 75, 82–85, 87) populations. Fourteen publications included participants with T1DM (59, 62–66, 68, 70, 71, 73, 74, 84, 85, 87), one publication included a sample of participants with T1DM or T2DM (75), one publication included participants with prediabetes or moderately controlled T2DM (83) and three publications included participants without diabetes (60, 61, 82). Publications included both controlled/inpatient (59, 62–65, 68, 74, 82, 85) and free-living settings (60, 61, 66, 70, 71, 73, 75, 83, 87).

In several aspects, there was substantial heterogeneity among the included publications. First, the number and type of performance metrics reported for the tested approaches differed substantially. Commonly reported performance metrics included the number of true and/or false positives and/or negatives (including frequencies per day and rates) (64, 66, 68, 70, 73, 75, 84, 85, 87), sensitivity (60, 61, 70, 73–75, 84, 87), specificity (60, 61, 70), accuracy (61, 70), precision (60, 61, 66), F1-score (60, 61, 66), Matthew’s correlation coefficient (60, 61), Pearson’s correlations (83), detection time or time until an insulin bolus was delivered (59, 62–66, 70, 73–75, 84), change in glucose concentrations (62, 65, 84, 85), and area under the curve (82). However, even when the same metrics were reported, their definition was sometimes inconsistent across publications. For instance, the detection window for true positive detections ranged from 60 to 180 min, depending on the publication (66, 73, 75). In addition, the general study setup varied between publications, including differences in the sample composition, the use of meal announcement/meal-accompanying insulin boluses, the ground-truth method used for identifying IA, the devices used, and the scope of the data collection (Table 1).

Table 2 shows the result of the critical appraisal of all included publications. There were some concerns regarding the applied methodology for all publications; these concerns were substantial for most publications. In 16/19 (84.2%) publications, the sample consisted exclusively of individuals with (pre)diabetes (59, 62–66, 68, 70, 71, 73–75, 83–85, 87). Further, 9/19 (47.4%) publications used error-prone methods for measuring the ground truth of IA, mostly self-reported IA (60, 61, 66, 70, 71, 73, 75, 83, 87). Moreover, 7/19 (36.8%) publications used meal announcements and/or meal-accompanying insulin boluses (66, 68, 70, 71, 73, 75, 87). Finally, 8/19 (42.1%) publications utilized other inputs besides CGM, e.g., heart rate or the insulin sensitivity factor (59–61, 63, 64, 70, 74, 82). Overall, 15/19 (78.9%) publications elicited methodological concerns in two or more appraised domains (59–61, 63, 64, 66, 68, 70, 71, 73–75, 83, 84, 87), and all publications had methodological concerns in at least one of the appraised domains (59–66, 68, 70, 71, 73–75, 82–85, 87).

**Table 2:** Critical appraisal of included publications.

Publication	Error-prone IA ground-truth method	Sample with (pre)diabetes	Meal announcement/insulin boluses	Other inputs in addition to CGM	Overall rating
Atlas et al. (59)	x	✓	x	✓	★★☆☆
Bertrand et al. (60)	✓	x	x	✓	★★☆☆
Bertrand et al. (61)	✓	x	x	✓	★★☆☆
Dassau et al. (62)	x	✓	x	x	★★★☆
Dovc et al. (63)	x	✓	x	✓	★★☆☆
El Fathi et al. (64)	x	✓	x	✓	★★☆☆
Faccioli et al. (66)	✓	✓	✓	x	★☆☆☆
Fushimi et al. (68)	x	✓	✓	x	★★☆☆
Godoy et al. (70)	✓	✓	✓	✓	☆☆☆☆
Hoyos et al. (71)	✓	✓	✓	x	★☆☆☆
Kölle et al. (73)	✓	✓	✓	x	★☆☆☆
Mosquera-Lopez et al. (74)	x	✓	x	✓	★★☆☆
Ornetzeder et al. (75)	✓	✓	✓	x	★☆☆☆
Palacios et al. (82)	x	x	x	✓	★★★☆
Palisaitis et al. (65)	x	✓	x	x^a^	★★★☆
Popp et al.(83)	✓^b^	✓	x	x	★★☆☆
Samadi et al.(84)	?	✓	x	x	★★☆☆
Turksoy et al. (85)	x	✓	x	x	★★★☆
Weimer et al. (87)	✓	✓	✓	x	★☆☆☆

x, no; ✓, yes;?, no information available; CGM, continuous glucose monitoring; IA, ingestive activity. “x” in all columns would signal minimal methodological concerns and thus an overall rating of four stars in the rightmost column; the more “✓,” the greater the methodological concerns, and consequently, the fewer stars are awarded in the rightmost column. Note that for the overall rating, no available information (“?”) was treated as eliciting methodological concerns (“✓”). ^a^While insulin pump data were also used in this paper, the meal detection algorithm relies only on glucose data as input. ^b^The authors state that self-reported IA is not seen as the ground-truth method in their work; however, in the absence of direct observation of IA in this study, herein, we assume self-reported IA as the ground-truth.

### 3.2 Overview of detection approaches

Our review identified a wide range of methods to automatically detect IA, including fuzzy logic (59, 63), model predictive control (74), support vector machine (60), random forest (60, 61, 82), (extreme) gradient boosting trees (60, 61, 82), backward difference (62), Kalman filter estimation (62), second derivative of glucose (62), Kalman filters (64, 68), switching signal generator (68), simulation-based explanation (83), classification of horizons (73), analysis of the glucose trajectory (84), pattern recognition using linear discriminant analysis (73), and threshold violation-based approaches (73). Further, adaptive model-based (64), super-twisting-based (66, 71), feedback scheme-based (70), and physiological parameter-invariant-based (87) meal detection approaches were applied.

The reviewed approaches used different inputs to automatically detect IA. As summarized in Table 2, some methods relied solely on CGM as an input (62, 65, 66, 68, 71, 73, 75, 83–85, 87). Others also included data from insulin treatment or other sensor systems (e.g., accelerometry, photoplethysmography, temperature sensors; see Table 1) (59–61, 63, 64, 70, 74, 82).

### 3.3 Performance of the approaches

We identified several CGM-based approaches for the automatic detection of IA that achieved high values in the respective performance metrics (Table 1). However, the substantial heterogeneity in the applied methodology and reporting of results needs to be considered.

For instance, Godoy and colleagues achieved 98.9% sensitivity, 96.7% specificity, and 97.6% accuracy with their feedback scheme-based algorithm (70). Notably, the algorithm uses certain patient-specific parameters, such as the insulin sensitivity factor derived from participants’ usual diabetes treatment (70). Similarly, the algorithm by El Fathi et al. successfully detected 12/12 meals without any false positives and a detection time of 35 min (64). In two publications using the same dataset, Bertrand et al. investigated several IA detection approaches in individuals without diabetes (60, 61). A range of performance metrics is reported in both publications. In the first publication, the highest achieved mean sensitivity was 66.8%, and the highest achieved mean specificity was 77.3%, for example (60). In the second publication, the highest achieved mean sensitivity was 66.8%, and the highest achieved mean specificity was 97.7% (61). Importantly, in both publications, the models did not exclusively rely on CGM as input (60, 61). Similarly, Palacios et al. had a sample of individuals without diabetes (82). However, their models, too, did not exclusively rely on CGM as input, but also utilized other physiological variables such as heart rate and skin temperature (82). Palacios et al. reported the area under the receiver operating characteristic curve (AUC-ROC) and the area under the precision-recall curve (AUC-PR) (82). For cold-start cases with a window size of *k* = 110 min, they reported an AUC-ROC of 89.1% and an AUC-PR value of 80.3% (82). For non-cold-start cases and *k* = 20 min, the AUC-ROC was 99.6%, and the AUC-PR was 96.4% (82).

The performance of CGM-only approaches, which hold particularly great value for practical applications, varied substantially. Sensitivities varied between 20.8% (73) and 96.8% (85). Where reported, average false positive detections ranged from 0.4 (75) to 2.8 (73) IA events per day. Selected publications further reported a false positive rate of 20.8% (84), a false discovery rate of 16.6% (74), and a median precision value of 73.0% (66). Moreover, publications reported detection times between 11.8 min (mean) (73) and 45.0 min (median) (66). Importantly, all CGM-only approaches were tested on samples consisting exclusively of individuals with (pre)diabetes, some of which also used meal announcements/insulin boluses. A detailed description of the performance metrics for each of the included publications is provided in Table 1.

### 3.4 Detection times

The detection time is the relevant metric to evaluate whether the identified CGM-based IA detection approaches could be used in the context of JITAIs.

A detection time measure was reported in 11/19 (57.9%) publications (59, 62–66, 70, 73–75, 84). The detection time was commonly defined as the time between the start of the IA (i.e., typically a meal) and its (automatic) detection by the CGM-based approach. Mean (59, 62, 64, 70, 73–75, 84) and median detection times (65, 66, 84) were reported, thus impeding direct comparisons. One publication reported the median time of the delivered prandial insulin boluses (63).

Overall, the reported detection times varied between 9.0 min (mean) (70) and 45.0 min (median) (66), with most values falling into the 20-to-40-min range (Table 1).

## 4 Discussion

The primary objective of this review was to examine whether CGM can be used to automatically detect IA in (near-)real time. In sum, there are various promising approaches that show satisfactory to excellent performance on measures such as sensitivity and specificity. However, the performance of CGM-based methods for automatically detecting IA varies. Similarly, detection times vary, but currently, they appear too long to administer JITAIs for acutely altering IA. Methodological issues and overall heterogeneity among publications make it difficult to recommend the best-performing approach.

### 4.1 Which approaches using CGM for the automatic detection of IA in (near-)real-time have been investigated, and have these approaches relied solely on CGM or also used other data (e.g., sensors/wearables)?

Our results indicate that both CGM-only approaches and those supplemented with other input data (e.g., accelerometry, photoplethysmography, temperature sensors) have been tested. Moreover, various algorithms have been used to detect IA. Since approaches using different sensor modalities and/or programming methods were successful at automatically detecting IA, it is evident that various solutions can be used for automated, CGM-based IA detection.

### 4.2 How accurate are these approaches in detecting IA?

Our review showed that the performance evaluation of any single approach depends on the respective case and priorities. For example, if the goal is to combine a CGM-based approach with smartphone prompts to enable comprehensive diet logs, the method should have high sensitivity to avoid missing a potential IA (false negative). In this case, specificity would only play a minor role as nothing is lost by sending a prompt in response to a false positive detection – the prompt can remain unanswered by the patient/participant. In contrast, when the goal is to use the CGM-based approach as a stand-alone IA assessment tool, high specificity would be critical to avoid artificial inflation of the number of daily meals, for example. Thus, a single best approach for all scenarios could not be identified. The substantial heterogeneity of the applied methods and reporting of results, including the broad range of the number and type of reported performance metrics and their varying definitions, made it difficult to compare the performance of the different approaches.

However, collectively, our results demonstrate that there are indeed several relatively well-performing CGM-based approaches for the automatic detection of IA. One example is the feedback scheme-based algorithm by Godoy et al., which achieved near-perfect sensitivity, specificity, and accuracy (70). However, this algorithm relies on several patient-specific parameters as input that are derived from participants’ usual diabetes treatment (70). Thus, it remains to be determined whether this approach could be adapted to work equally well in individuals without diabetes, for whom these data are not routinely assessed. Similarly, methodological issues further limiting studies’ internal and/or external validity pertain to using meal announcements or insulin boluses and focusing on samples with diabetes in the reviewed studies. All included publications suffered at least one such methodological limitation (Table 2).

Several reviewed articles reported solutions that relied solely on CGM as input for their IA detection algorithms. Performance among these approaches varied, but sensitivities ≥90% were achieved by several groups (73, 84, 85), and false positive occurrences < 1 per day were reported (75). This suggests that inputs other than CGM are not necessary to achieve excellent performance in automatically detecting IA.

Of note, some algorithms that incorporated inputs other than CGM might also work with CGM as their only input for the specific goal of IA detection. For example, in Bertrand et al.’s machine-learning algorithms, data from two wrist-worn activity trackers were incorporated in addition to the CGM data (61). However, the 20 most important features were derived from the CGM data (61). Hence, it is likely that an adaptation of their algorithm that relies exclusively on the CGM data as input might also achieve good – albeit likely *somewhat* worse – IA detection performance. Similar cases can be made for other publications in which insulin data were used as input in addition to the CGM data (59, 63, 64). These results suggest that it is possible to automatically detect IA using CGM-based and even CGM-only algorithms.

### 4.3 Can these approaches be used in the context of JITAIs?

Generally, to successfully administer a dietary JITAI, IA must be detected in (near-)real-time. However, precisely how short the detection would have to be depends on the specific goal, as outlined before. Detection times as fast as 9.0 min were reported (70), but most approaches needed 20 to 40 min to detect IA (Table 1). This can generate feedback on IA much faster than traditional dietary assessment methods, such as 24-h recalls, thus creating opportunities for earlier intervention. For instance, detecting deviations from a standardized study procedure (e.g., when IA is detected in a fasting window) is likely possible. Further, when deviations from a specific meal plan (e.g., low carbohydrate) are detected, the plan could be adjusted for the subsequent meals on the same day. Automated meal detection could further trigger behavioral intervention prompts regarding portion size and eating rate (i.e., reminders to eat more slowly) for future meals. However, in most cases, detection times are too long to modify/influence IA truly in the moment it occurs (e.g., a participant on a ketogenic diet has likely already finished a carbohydrate-rich meal by the time it is detected). Regardless, it is debatable if that is really the goal and what intervening *during* an eating event would look like.

### 4.4 Implications for clinical and research practice

Our review shows that several CGM-based options for the automatic detection of IA exist. Ultimately, the specific use case will dictate the most suitable approach. Different approaches might be appropriate depending on factors such as the budget, population, targeted level of wearing comfort, and goal of the automatic IA detection.

Notably, other innovative methods for the automatic detection of IA, such as those using wearable-, sensor-, and image-based methods (9, 15–18), are also promising. These methods may even be superior to CGM-based approaches regarding detection times. Wang and colleagues identified several devices that can quickly detect IA (16), such as a headband device that can detect eating events via chewing sounds within only 3 min (16, 90). Similarly, a pilot study by Kumar and colleagues investigating the use of abdominal sounds to detect IA found an average detection time of only 4.3 min (91). It has even been demonstrated that eating events can be predicted ahead of time (16, 92). Yang et al. used a camera, a GPS device, and an accelerometer to predict eating and food-purchasing events up to 4 min in advance (92). The authors found that a trained gradient-boosting model achieved a mean accuracy of 72.9% in predicting eating events 0–4 min in advance (92). This highlights that different methodologies might have inherent strengths and limitations. The suboptimal detection times might be considered an inherent limitation of CGM-based approaches. Recent advances have tried to solve the CGM-inherent lag time issue (93), but more research is needed. It remains to be seen whether these limitations inherent to using CGM for automatically detecting IA can be overcome. On the other hand, one key benefit of using CGM might be its unobtrusiveness, which could facilitate its acceptance in practice. This unobtrusiveness contrasts many other, more obtrusive approaches such as glasses and camera-based methods (9, 16, 18).

A promising prospect might be to use a sensitive CGM-based approach that sends a prompt to the patient/participant asking them to log IA in case of a true positive detection. Thus, the CGM-based approach would serve as an automated reminder. That way, a false positive detection does not automatically lead to erroneous IA information but needs to be verified by the person. In this context, the suboptimal detection times also likely would be acceptable.

### 4.5 Limitations and directions for future research

#### 4.5.1 Sample characteristics

Unsurprisingly, most publications included samples with diabetes, as the primary use case for automated IA detection is AP systems. However, to examine the potential of CGM-based approaches for detecting IA in various populations, more research in more diverse populations, including healthy individuals, should be conducted. This is particularly important as the generalizability of previous findings to non-diabetic individuals is likely limited, for instance, due to the usually far lower variations in blood glucose levels in persons without diabetes (77) as compared to persons with diabetes (78). Thus, there may likely be systematic differences in the performance of such approaches in individuals with diabetes compared to those without diabetes. Moreover, in many studies, meals were announced to the system, and/or manual insulin boluses accompanied the registered meals. For example, Ornetzeder and colleagues evaluated the detection performance of three previously published algorithms (79–81) using meals accompanied by insulin boluses (75). While the resulting performance metrics of this publication and similar others are promising, they need to be interpreted considering the applied insulin boluses. Ornetzeder et al. argue that this potential distortion was deemed acceptable due to a lack of alternative, insulin bolus-free datasets and the time it takes for the administered insulin to achieve its peak action (75). However, it is still possible that the results of CGM-based IA detection approaches might differ in scenarios without exogenous insulin infusions. Specifically, the administered insulin might flatten the blood glucose excursions from the meal’s start, making its automatic detection less likely. In line with this, Faccioli et al. state that some of their false negatives might have been related to the attenuated postprandial CGM curves following the administration of meal-accompanying insulin therapy (66). At the same time, it should be considered that the postprandial glucose excursions of individuals with insulin-dependent diabetes would be much more pronounced without insulin treatment than in non-diabetic individuals (82). As such, it could be argued that by administering meal boluses, the postprandial glucose excursions of individuals with diabetes more closely approximate those of individuals without diabetes. Direct evidence is, of course, still necessary to increase confidence in any conclusions. Thus, future studies should ultimately enroll more individuals without diabetes.

#### 4.5.2 Research focus

Moreover, it also needs to be considered that for the initialization of closed-loop systems, background information (e.g., treatment management, physical characteristics of the patient) is typically provided to the system (59). This information may only sometimes be readily available in other contexts. In addition, the goals of algorithms geared toward use in closed-loop/AP systems might differ from approaches aimed at the use for automatic detection of IA in general. For instance, in their AP-oriented work, Kölle et al. focused on glucose excursions caused by larger meals because smaller meals or snacks, which do not cause a substantial increase in blood glucose levels, do not necessarily need to be detected and trigger an insulin bolus to ensure adequate glucose control (73). Yet, in a scenario where the automatic detection of IA via CGM is meant to provide information on *any* IA – irrespective of its size – this argument does not hold up. This example highlights the potential differences in the setup of algorithms depending on the goal.

Taken together, fundamentally different circumstances and goals may be pursued, and thus, algorithms may be constructed differently, depending on the research question. Consequently, it might be possible to further optimize algorithms to automatically detect IA in research or clinical settings other than closed-loop/AP systems.

#### 4.5.3 Comparability of approaches

There was substantial heterogeneity in how the performance of the investigated approaches was evaluated across the reviewed publications. Thus, as noted by others (66), a direct comparison between the approaches is difficult due to differences in the utilized datasets, preprocessing, and evaluation methods. Differences like these ultimately hamper the search for the best-performing approaches. Performance metrics reported in publications should include at least the following measures: the number of true positives, false positives, and false negatives, which can be used to calculate important metrics such as sensitivity and precision; the detection time, defined as the time from the start of the IA to the time the algorithm detects the IA, whose reporting allows researchers and practitioners to judge whether a specific approach could be used to administer JITAIs, for example. A short detection time followed by a prompt could also allow for more immediate self-reported IA. More accurate self-reports could be the consequence due to diminished recall bias.

#### 4.5.4 Future avenues

In general, more research should be dedicated to using CGM for the specific goal of automatically detecting IA in a broad range of populations, particularly in individuals without diabetes. Such approaches have several potential benefits, but prior research has mainly focused on using CGM for diabetes care and AP systems. However, as explained, algorithms will likely be constructed differently for the specific goal of automatically detecting IA. Moreover, previous findings will have to be replicated and extended in non-diabetic samples to overcome the currently limited generalizability.

Depending on the use case, several advancements would be necessary to rely exclusively on a CGM-based/CGM-only approach for the remote monitoring of IA. To fully automate the logging of IA times in a reliable manner, most systems would have to be even more accurate than they currently are.

If the goal is to further automate IA timing and effectively log macronutrient intake, approaches would have to incorporate specific algorithms for this task. Several publications explored whether estimating macronutrients from CGM data is possible. For instance, Samadi et al. estimated the carbohydrate content of meals (84). Results were promising, with 64.1% of the detected IA events having an absolute carbohydrate estimation error of less than 25 g (84).

Similarly, if the goal is to administer JITAIs to impact acute IA, detection times would have to decrease further. However, as mentioned above, the lag time-caused suboptimal detection times might have to be considered an inherent limitation of CGM-based approaches. Only if future studies succeed at further reducing detection times will the application of CGM-based approaches for dietary JITAIs aiming to alter IA in the moment in the truest sense of the word become possible. This is especially true for cases in which meals are followed by only small and/or delayed postprandial glucose excursions (e.g., after high-fat meals) or when meals contain only a small amount of carbohydrates, as (timely) detection appears difficult here (66, 74). It would also be necessary to explicitly test the detection performance in cases of such challenging IA (e.g., ketogenic diets). Empirical data on such cases might enable the prediction of in which settings CGM-based approaches can be used for successfully detecting IA (e.g., only in contexts where at least moderate amounts of carbohydrates are consumed).

We advise that future studies use different approaches on the same dataset, providing comprehensive CGM and objective IA data, and then compare their performance using the abovementioned metrics. A starting point could be to compare the CGM-only approaches highlighted in Table 2. Such a fair and standardized comparison could further illuminate the currently most promising approach(es).

While CGM-only approaches are highly attractive because they only necessitate one single sensor (i.e., the CGM), multi-sensor solutions also hold great potential and should thus be further investigated. Specifically, combining the strengths of different sensors (e.g., CGM and wristbands) may yield superior results as compared to relying on only one sensor, although this remains to be determined empirically.

Lastly, similar to others (10), we strongly advise that researchers use interdisciplinary collaborations to develop new CGM-based dietary monitoring tools to combine technological and biological/nutritional expertise. Interdisciplinary collaborations should ensure that the resulting tools are useful and optimized from both perspectives.

### 4.6 Conclusion

Based on an exhaustive and systematic literature search, this scoping review shows that it is possible to automatically detect IA using CGM-based approaches. Despite methodological issues and substantial overall heterogeneity among publications, CGM-based dietary monitoring might complement clinical and research practice.

## Data availability statement

The original contributions presented in the study are included in the article/supplementary material, further inquiries can be directed to the corresponding author.

## Author contributions

JB: Conceptualization, Methodology, Writing – original draft, Writing – review & editing, Visualization. CG: Methodology, Writing – review & editing. SHF: Methodology, Writing – review & editing. KK: Conceptualization, Writing – review & editing. CH: Conceptualization, Methodology, Writing – original draft, Writing – review & editing.
